# Natural products of medicinal plants: biosynthesis and bioengineering in post-genomic era

**DOI:** 10.1093/hr/uhac223

**Published:** 2022-09-28

**Authors:** Li Guo, Hui Yao, Weikai Chen, Xumei Wang, Peng Ye, Zhichao Xu, Sisheng Zhang, Hong Wu

**Affiliations:** Shandong Laboratory of Advanced Agricultural Sciences at Weifang, Peking University Institute of Advanced Agricultural Sciences, Weifang, Shandong 261000, China; Key Laboratory of Bioactive Substances and Resources Utilization of Chinese Herbal Medicine, Institute of Medicinal Plant Development, Chinese Academy of Medical Sciences & Peking Union Medical College, Beijing 100193, China; Shandong Laboratory of Advanced Agricultural Sciences at Weifang, Peking University Institute of Advanced Agricultural Sciences, Weifang, Shandong 261000, China; School of Pharmacy, Xi’an Jiaotong University, Xi’an 710061, China; State Key laboratory for Conservation and Utilization of Subtropical Agro-bioresources, Guangdong Laboratory For Lingnan Modern Agriculture, College of Life Sciences, South China Agricultural University, Guangzhou 510642, China; College of Life Science, Northeast Forestry University, Harbin 150040, China; State Key laboratory for Conservation and Utilization of Subtropical Agro-bioresources, Guangdong Laboratory For Lingnan Modern Agriculture, College of Life Sciences, South China Agricultural University, Guangzhou 510642, China; State Key laboratory for Conservation and Utilization of Subtropical Agro-bioresources, Guangdong Laboratory For Lingnan Modern Agriculture, College of Life Sciences, South China Agricultural University, Guangzhou 510642, China

## Abstract

Globally, medicinal plant natural products (PNPs) are a major source of substances used in traditional and modern medicine. As we human race face the tremendous public health challenge posed by emerging infectious diseases, antibiotic resistance and surging drug prices etc., harnessing the healing power of medicinal plants gifted from mother nature is more urgent than ever in helping us survive future challenge in a sustainable way. PNP research efforts in the pre-genomic era focus on discovering bioactive molecules with pharmaceutical activities, and identifying individual genes responsible for biosynthesis. Critically, systemic biological, multi- and inter-disciplinary approaches integrating and interrogating all accessible data from genomics, metabolomics, structural biology, and chemical informatics are necessary to accelerate the full characterization of biosynthetic and regulatory circuitry for producing PNPs in medicinal plants. In this review, we attempt to provide a brief update on the current research of PNPs in medicinal plants by focusing on how different state-of-the-art biotechnologies facilitate their discovery, the molecular basis of their biosynthesis, as well as synthetic biology. Finally, we humbly provide a foresight of the research trend for understanding the biology of medicinal plants in the coming decades.

## Natural products in medicinal plants: hidden treasures with healing power

**Figure 1:**
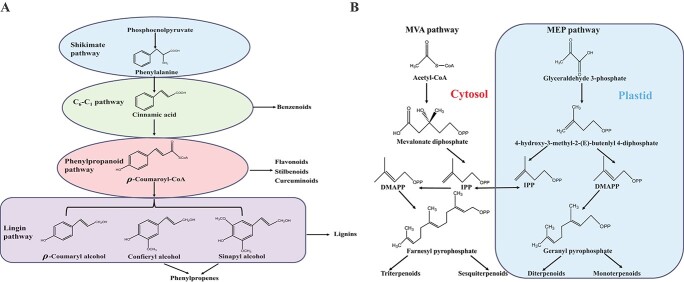
Proposed general biosynthetic pathway of plant phenolics (**A**) and terpenoids (**B**). DMAPP: dimethylallyl pyrophosphate; IPP: isopentenyl pyrophosphate; MEP: methylerythritol phosphate; MVA: mevalonic acid.

**Figure 2:**
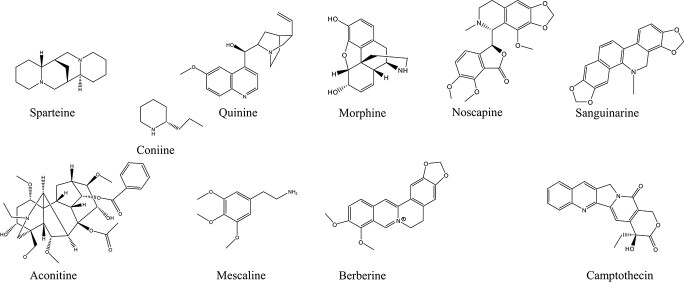
Common alkaloid types (**A**) and examples of pharmaceutical alkaloids (**B**) in medicinal plants.

Plants have existed on Earth for hundreds of millions of years and evolved ingenious chemical factories to survive exogenous and endogenous stresses [1]. These chemicals known as secondary metabolites or natural products are synthesized by plants to accommodate environmental changes without disrupting much of their cellular and developmental physiological processes [2]. To date more than 100 000 natural products are present in the Kingdom of Plants, primarily involved in plant defense against biotic and abiotic stresses [3]. These powerful substances are also important chemical signals mediating plant communication with symbiotic microorganisms, and attracting pollinators and seed dispersal. Derived from primary metabolites, secondary metabolites accumulate at cellular, tissue and organ levels through diverse biosynthetic pathways [4]. Plant natural products (PNPs) are generally divided into three classes: phenolics, terpenoids, and alkaloids [5] and broadly used as pharmaceuticals, nutraceuticals, cosmetics, and fine chemicals. Phenolics are synthesized from the shikimic acid biosynthetic pathway where the final products are formed after phenylalanine and aromatic amino acids undergo deaminization, hydroxylation, and coupling reactions [7] (Fig. 1A). Ingeneral, phenolics consist of monophenols such as benzenoids,and polyphenols such as flavonoids, stilbenoids, and coumuminoids [6]. Terpenoids are biosynthesized through mevalonic acid and methylerythritol phosphate pathways from isopentenyl diphosphate (IPP, C_5_), the precursor and fundamental structural unit of all terpenoids including monoterpenoids (C_10_), sesquiterpenoids (C_15_), diterpenoids (C_20_), and triterpenoids (C_30_) [8] (Fig. 1B). Alkaloids are a large group of plant nitrogen-containing compounds with a broad range of pharmaceutical activities such as painkilling (e.g. morphine), cough-suppressing (e.g. noscapine), anti-inflammation (e.g. sanguinarine, berberine), and anti-cancer (e.g. vinblastine, noscapine) (Fig. 2). Originated from the amino acid and isoamylene biosynthetic pathway, alkaloids are generally classified into sparteine, quinine, mescaline, coniine, and aconitine (Fig. 2). The biosynthetic pathways of different alkaloids are diversified and often independent. Recently, Lichman [9] has summarized alkaloid pathways as four general steps: (i) accumulation of an amine precursor; (ii) accumulation of an aldehyde precursor; (iii) formation of an iminium cation; and (iv) a mannich-like reaction.

PNPs are bioactive substances dispensable to normal plant cellular functions yet vital to biodefense and environmental adaptation of plants with a sessile lifestyle. As a major source of traditional and modern medicine, PNPs have had broad implications for human health as a herb remedy for thousands of years, and changed the course of human civilization and history [9]. The healing power of medicinal herbs has long been recognized and harnessed by our human ancestors and forefathers, who learned to use plant-based folk medicine to cure ailments such as headache, fever, and pains. For example, archaeologists found in a grave from Shanidar – an archaeological site in Iraq – that a Neanderthal man may have used plant-based medicine at around 60 000 bce, the earliest record of human use of herb medicine [10]. In addition, ancient Europeans had started to cultivate and use different varieties of opium poppy plants at least 5000 years ago [11]. Another example is tobacco, which was historically used to treat various ailments including yaws, syphilis, and black death given its strong antimicrobial activities [12,13]. Much of early human knowledge about medical herbs is documented in ancient scriptures and literature such as *Treatise on Cold Pathogenic and Miscellaneous Diseases* by Zhang Zhongjing (Eastern Han dynasty) and *Compendium of Materia Medica* composed by Li Shizhen (Ming dynasty). However, the use of medicinal herbs in traditional medicine has been largely considered as empiricism with little knowledge of the chemical properties of the effective PNPs. The first isolated PNP was morphine, a benzylisoquiloine alkaloid in opium poppy (*Papaver somniferum*) plants, by Germany pharmacist Friedrich Sertürner at around 1817, marking the birth of modern chemistry of natural products [14]. Later on, another alkaloid quinine isolated from the bark of cinchona tree (*Cinchona officinalis*) became the first effective medicine against malaria, caused by mosquito-transmitted *Plasmodium* species [15]. Salicin, the original source of aspirin and identified in the bark of willow tree (*Salix babylonica*), is another significantly used PNP, used prominently as a pain reliever [16]. To date, hundreds of plant-based bioactive compounds have been identified and many are used as an effective treatment of human diseases such as ginsenoside (anti-tumor), paclitaxel/taxol (anti-tumor), and artemisinin (anti-malaria) etc. The sesquiterpene endoperoxide artemisinin from *Artemisia annua* is recommended by the WHO as the most effective drug against malaria [17]. The paclitaxel (taxol) isolated from the tree barks of *Taxus* genus has been approved for the treatment of ovarian, breast, and lung cancer, as well as Kaposi’s sarcoma [18]. These examples have demonstrated that medicinal plants and their powerful natural products have great healing power and have shaped the development of human history.

Throughout history, the human race has battled with many infectious diseases such as tuberculosis, cholera, malaria, black death, influenza, smallpox, etc. The most recent public health challenge comes from the coronavirus pandemic initiated in 2019 (COVID-19) which continuously threatens the world with the non-stop emergence of new variants. These pandemics each and collectively have led to significant progress in human knowledge of medical sciences and the generation of new public health solutions in which medicinal plants played important roles. Because antibiotics that target bacterial and fungal pathogens are futile against viral infection, the current solution against viral infections such as influenza and coronaviruses is primarily through vaccination, whereas effective virus-killing drugs are highly desired but scarce. Whereas western medicine working against COVID remains under development and clinical trials such as sabizabulin [19], remdesivir [20], hydroxychloroquine [21], and PNPs with anti-viral activities have been reported as effective to contain viral replication and alleviate patient symptoms. For example, flavonoids such as neo-hesperidin, hesperidin, baicalin, kaempferol 3-*O*-rutinoside, and rutin from different sources, and a series of xanthones from *Swertia* plants could effectively interact with SARS-CoV-2 targets [22]. In addition, molecular docking was recently performed using flavonoids from fruit peel of *Citrus reticulata* ‘Chachi’ to target the spike proteins, 3CLpro, PLpro, and RdRp of SARS-CoV-2, suggesting that many flavonoids have stronger affinity with the targets than do positive control drugs [23]. Recently, in a multicenter, prospective and randomized controlled clinical trial, Lianhuaqingwen capsule, a manufactured product of the traditional Chinese medicine (TCM) formula, could significantly inhibit SARS-CoV-2 replication [24]. Despite the promising therapeutic effect of medicinal herbs against COVID, it remains unknown what is the causing substance, and mechanisms of action against the virus. Furthermore, because biosynthetic pathways of most PNPs are elusive, the working molecules cannot be obtained in sufficient quantities to satisfy the need for drugs to control global pandemics. For decades, the biochemical pathways of PNP biosynthesis, genome architecture, and regulation of PNP production, and most importantly bioengineering to massively produce bioactive PNPs are all extensively researched areas in medicinal plant biology and chemistry. Here, we attempt to review the current research of PNPs by focusing on how different state-of-the-art multi-discipline technologies facilitate their discovery, the molecular basis of biosynthesis, as well as bioengineering via breeding and synthetic biology. Finally, we highlight a few research trends regarding the exploitation of medicinal plants in the next decades [25].

## Decoding biosynthetic pathways of PNPs is key to their applications

The challenge of acquiring PNPs *en masse* stems from the fact that medicinal plants have a tight regulation of producing these chemicals, mostly localized in specific cells and tissues under certain conditions [26]. The biosynthesis of PNPs typically goes through a cascade of enzymatic reactions converting primary metabolites into various structurally diverse secondary metabolites. Although some reactions can occur spontaneously in nature, most steps require a catalysis by enzymes such as cytochrome P450, methyltransferases, O-methyltransferases, deaminases, UDP-glucuronosyltransferases, etc. A major challenge in exploitation of PNPs is to understand their biosynthetic pathways so that the bioengineering approach can be applied to produce them on a massive scale through plant breeding or synthetic biology. Fully resolving the biosynthetic pathways of any PNP is a daunting task, because plants have evolved a complex cellular network of metabolic pathways [27], formed by various enzymes catalyzing a myriad of biochemical reactions, and regulatory proteins fine-tuning the spatial and temporal accumulation of PNPs. Currently, the full biochemical pathways remain unknown for the majority of medicinal PNPs after years of research efforts, highlighting the difficulty of decoding PNP biosynthetic pathways.

Before the genomic era, plant biosynthetic pathway characterization was laborious and time-consuming, either relying on approaches such as isotope labeling and forward genetics, such as by creating random mutants followed by analysing their metabolic profiles, or involving sequence-homology based gene cloning to identify individual biosynthetic enzymes [28]. These studies typically identify genes encoding parts of the pathways through guilt-by-association, as loss- or gain-of-function mutations of true biosynthetic genes can alter metabolic profiles. However, they are usually inept to resolve the complete components and reconstruct the pathway owing to the pleiotropic or promiscuous nature of the biosynthetic enzymes. The majority of our knowledge about PNP biosynthetic pathways so far is derived from such homology-based or transcriptome-based gene mining [29, 30]. With growing volumes of genomic data available for medicinal plants, it is now common to exploit high-throughput data mining combined with experimental validations to untangle the complex biosynthetic pathways and networks underlying PNP accumulation in medicinal herbs.

PNP biosynthetic genes are usually co-expressed and co-regulated in specific tissues and growth stages. Therefore, in the post-genomic era, gene expression profiling techniques such as whole transcriptome sequencing (RNA-seq) enable researchers to quickly narrow down the co-expressed candidate genes encoding specific biosynthetic pathways, typically via comparative transcriptiome analysis of plant samples with contrasting levels of metabolic production. Transcriptomic analysis combined with pathway inference based on chemistry logic, and experimental validations in heterologous hosts are routinely used to identify biosynthetic genes for PNPs such as thebaine and noscapine of opium poppy (*P. somniferum*) [31], sanguinarine and chelerythrine of *Macleaya cordata* [32], vinblastine of *Madagascar periwinkle* [33], colchicine of *Colchicum autumnale* [34] and strychnine of *Strychnos nux-vomica* [35]. Alternatively, proteomic profiling is also used to identify proteins corresponding to specific biosynthetic pathways, capturing translational and post-translational modifications unseen in transcriptomic data. Candidate genes identified by omic profiling analysis are then validated experimentally to ascertain their biochemical functions such as catalyzing specific reactions, metabolite transport or transcriptional regulation. It typically involves heterologously expressing the candidate gene(s) in microbial cells including bacteria (*Escherichia coli* [36]*, Corynebacterium glutamicum* [37]), yeasts (*Saccharomyces cerevisiae* [38], *Pichia pastoris* [39]), or plant chassis such as tobacco (*Nicotiana benthamiana* [40]) and algae (*Chlamydomonas reinhardtii* [41], *Phaeodactylum tricornutum* [42]), followed by detection of the target metabolites using untargeted metabolomics. Metabolomics study the full complement of plant metabolites through high performance liquid chromatography (HPLC)- or gas chromatography (GC) coupled with mass spectrometry (MS). Integrated mining of multi-dimensional data such as transcriptomic or proteomic with metabolomic profiling in different tissues and growth stages of medicinal plants enables gene-metabolite association. Network mining using these big data can reconstruct the gene co-expression networks correlated with accumulation of PNPs, thus generating hypothesis for downstream experimental validations [43]. This approach still suffers from the fact that many biosynthetic genes may be cryptic or lowly expressed, leaving them undiscovered by the expression-based methods. Thus, systematic decoding of PNP biosynthetic pathways will require a framework of high-throughput analysis of omic data from plants growing under multiple conditions or developmental stages. For example, two recent studies acquired transcriptomic and metabolomic data of tomato and rice plants across the full spectrum of growing stages, and by integrative data analysis revealed gene modules associated with natural products [44, 45]. It is expected that similar analysis from full growth stages of medicinal plants will facilitate discovery of biosynthetic genes and yield critical insight into the regulatory networks underlying PNP biosynthesis.

## Medicinal plant genomes yield insights into composition and evolution of biosynthetic pathways

Genome sequence dictates the foundation of biological functions of all life forms. Despite the progress made by the homology- and transcriptome-based approach, the elucidation of full biosynthetic pathways is often hampered by a lack of reference genome sequences for medicinal plants. A reference genome sets the foundation to identify all protein-coding genes, regulatory DNA elements and importantly their precise genomic locations. Next-generation sequencing (NGS) and third-generation sequencing (TGS) technologies have revolutionized biological sciences by changing the way genomes are decoded. The first human and plant (*Arabidopsis thaliana*) genome assembly were initially achieved using Sanger sequencing. Despite the high accuracy, Sanger sequencing is expensive and time-consuming to generate sequencing data for assembly of eukaryotic genomes of mid to large sizes. Since around 2005, genome assembly projects started to adopt high-throughput sequencing platforms like Solexa, Ion Torrent and later Illumina, giving rise to the first reference genomes for many organisms. However, contig-level assemblies using short reads have low contiguity (low N50) with numerous assembly errors due to high repeat content of plant genomes, even when a high coverage and long insert such as mate-pair libraries or linked-reads are used. Scaffold level assemblies are often improved using genetic map data such as GBS (genotyping by sequencing), or optical maps to anchor the contigs to linkage groups. However, a high-resolution genetic map is essential to reducing contig misplacement but often unavailable for non-model plants. TGS technologies developed by Pacific Biosciences (PB) and Oxford Nanopore technology (ONT) produce single-molecule DNA sequencing reads of 20 kb or longer, albeit error-prone (up to 15%). TGS became a game changer for genome assembly because long reads can often span most repetitive regions. Besides, technologies such as chromatin conformation capture sequencing (e.g. Hi-C) and Bionano are now routinely used to anchor contigs to chromosomes and correct misassemblies present in NGS and TGS draft assemblies. As a result, for model organisms and many agricultural organisms, genome assembly quality and contiguity have leaped to much higher levels with a combination of long-read and short-read sequencing data.

Medicinal plant genomes have a wide genome size range, high heterozygosity rates and repeat contents, making them difficult to assemble correctly [46]. Leveraging these different technologies combined with improving bioinformatic algorithms has yielded a growing number of high-quality medicinal plant genome assemblies and annotations. Nearly 100 species of medicinal plants (Table 1) have at least one version of reference genome available [47–139], although the quality of current genome assemblies varies depending on the genome complexity of medicinal plants and choice of sequencing technologies as well as computational tools used in assembly. The herbgenomics initiative, firstly proposed in 2010, has greatly promoted the elucidation of biosynthetic pathways for many medicinal bioactive ingredients [48]. Under this initiative and many independent genome projects, several medicinal plants have had reference genomes assembled even at chromosome level, such as *P. somniferum* [49], *Camptotheca acuminata* [50], *Scutellaria baicalensis* [51], *Panax notoginseng* [52], *Tripterygium wilfordii* [53], *Salvia miltiorrhiza* [54], *Taxus wallichiana* [55], and *Erigeron breviscapus* [56]. Lately, a Chinese consortium of the 1 K Herb Genomes Project has been officially launched to produce high-quality genome sequences for 1000 high-value TCM in order to promote the study and exploitation of their PNPs.

**Table 1:** Sequenced genomes of medicinal plants and their primary medicinal ingredients.

**Species**	**Genome size**	**Contig N50**	**No. of annotated genes**	**AssemblyLevel** ^**a**^	**Release time**	**Primary medicinal ingredients**	**Reference**
*Apium graveolens*	3.33 Gb	790.6 kb	31 326	C	2020	Apigenin and luteolin	[60]
*Aralia elata*	1.05 Gb	1.20 Mb	35 042	C	2022	Oleanane-type triterpenoids	[61]
*Coriandrum sativum*	2.12 Gb	604.1 kb	40 747	C	2020	Mannitol, furfural and linalool	[62]
*Panax ginseng*	3.41 Gb	19.75 Mb	65 913	C	2022	Dammarane-type saponins	[63]
*Panax japonicus*	2.09 Gb	1.22 Mb	74 307	C	2022	Dammarane-type saponins	[63]
*Panax notoginseng*	2.66 Gb	1.12 Mb	37 606	C	2020	Dammarane-type ginsenoside	[52]
*Panax quinquefolius*	3.60 Gb	0.87 Mb	64 247	C	2022	Dammarane-type saponins	[63]
*Panax stipuleanatus*	2.15 Gb	2.88 Mb	41 224	C	2022	Dammarane-type saponins	[63]
*Allium sativum*	16.24 Gb	194 kb	57 561	C	2020	Allicin	[64]
*Dendrobium huoshanense*	1.28 Gb	598 kb	21 070	C	2020	Polysaccharides and alkaloids	[65]
*Dendrobium officinale*	1.23 Gb	1.44 Mb	27 631	C	2021	Polysaccharides, alkaloids and flavonoids	[66]
*Gastrodia elata*	1.04 Gb	9.18 Mb	18 844	C	2022	Gastrodin, phydroxybenzyl alcohol and parishin	[67]
*Arctium lappa*	1.73Gb	74.69 Mb	47 055	C	2022	Arctigenin and arctiin	[68]
*Artemisia annua*	1.55 Gb	262.1 kb	54 347	C, H	2022	Artemisinin	[69]
*Artemisia argyi*	8.03 Gb	6.25 Mb	279 294	C, H	2022	Hispidulin, jaceosidin, and eupatilin	[70]
*Carthamus tinctorius*	1.06 Gb	21.23 Mb	33 343	C	2021	Linoleic acid and hydroxysafflor yellow A	[71]
*Chrysanthemum nankingense*	2.53 Gb	130.7 kb	56 870	S	2018	Flavonoids and terpenes	[72]
*Erigeron breviscapus*	1.43 Gb	140.95 kb	43 514	C	2020	Scutellarin	[56]
*Smallanthus sonchifolius*	2.72 Gb	87.39 Mb	89 960	C	2022	Saccharides (inulin)	[68]
*Platycodon grandiflorus*	622.86 Mb	29.34 Mb	22 358	C	2022	Triterpenoid saponins	[73]
*Isatis indigotica*	293.88 Mb	1.18 Mb	30 323	C	2020	Indole alkaloids, β-sitosterol and daucosterol	[74]
*Lepidium meyenii*	743 Mb	81.78 kb	96 417	S	2016	Macaridine, macamides and macaene	[75]
*Polygonum cuspidatum*	2.56 Gb	2.77 kb	55 075	S	2019	Stilbenes and quinones	[76]
*Tripterygium wilfordii*	348.38 Mb	4.36 Mb	28 321	C	2020	Triptolide	[53]
*Ceratophyllum demersum*	860.5 Mb	2.57 Mb	30 138	C	2020	Plastocyanin and ferredoxin	[77]
*Camptotheca acuminata*	414.95 Mb	1.47 Mb	27 940	C	2021	Camptothecin	[50]
*Benincasa hispida*	912.95 Mb	145 kb	27 467	C	2019	Vitamins and flavonoids	[78]
*Gynostemma pentaphyllum*	518.46 Mb	6.67 Mb	25 285	C	2021	Gypenosides	[79]
*Luffa cylindrica*	656.19 Mb	8.80 Mb	25 508	C	2020	Sapogenins	[80]
*Momordica charantia*	293.6 Mb	3.3 Mb	26 427	C	2020	Cucurbitacins and cucurbitane glycosides	[81]
*Siraitia grosvenorii*	467.07 Mb	433.68 kb	30 565	S	2018	Mogrosides	[82]
*Lonicera japonica*	843.2 Mb	2.1 Mb	33 939	C	2020	Luteolin and chlorogenicacid	[83]
*Diospyros lotus*	907 Mb	1.06 Mb	40 532	C	2020	Tannin	[84]
*Camellia sinensis*	3.02 Gb	19.96 kb	36 951	C	2017	Caffeine and theanine	[85]
*Glycyrrhiza uralensis*	378.86 Mb	7.32 kb	38 135	S	2017	Glycyrrhizin and liquiritin	[86]
*Senna tora*	526.36 Mb	4.03 Mb	45 268	C	2020	Anthraquinones	[87]
*Spatholobus suberectus*	798.47 Mb	2.05 Mb	31 634	C	2019	Eriodictyol and dihydroquercetin	[88]
*Eucommia ulmoides*	947.84 Mb	13.16 Mb	26 001	C	2020	Pinoresinol diglucoside and aucubin	[89]
*Calotropis gigantea*	157.28 Mb	48.58 kb	18 197	S	2018	Cardenolides	[90]
*Catharanthus roseus*	523 Mb	26.2 kb	33 829	S	2015	Vinblastine and vincristine	[33]
*Gelsemium elegans*	335.13 Mb	10.23 Mb	26 768	C	2020	Gelsemine and koumine alkaloids	[91]
*Gardenia jasminoides*	535.68 Mb	1.03 Mb	35 967	C	2020	Genipin and crocins	[92]
*Morinda officinalis*	484.85 Mb	4.21 Mb	27 698	C	2021	Anthraquinones and monotropein	[93]
*Ophiorrhiza pumila*	439.90 Mb	18.49 Mb	32 389	C	2021	Camptothecin	[94]
*Andrographis paniculata*	284.32 Mb	5.15 Mb	24 015	C	2020	Neoandrographolide	[95]
*Mentha longifolia*	353 Mb	4.47 kb	35 597	S	2017	Menthone and menthol	[96]
*Ocimum basilicum*	2.06 Gb	48.30 Kb	78 990	S	2020	Linalool and cineole	[97]
*Origanum vulgare*	630.04 Mb	26.28 kb	32 623	S	2020	Carvacrol, thymol and ocimene	[97]
*Perilla frutescens*	1.24 Gb	3.21 Mb	93 008	C	2021	Perillaldehyde, perillaalcohol and limonene	[98]
*Pogostemon cablin*	1.94 Gb	7.97 Mb	109 696	C, H	2022	Patchouli alcohol	[99]
*Rosmarinus officinalis*	1.01 Gb	21.82 kb	51 389	S	2020	Rosmarinic acid, camphor and carnosol	[97]
*Salvia bowleyana*	462.44 Mb	1.18 Mb	44 044	C	2021	Tanshinone and salvianolic acid	[100]
*Salvia miltiorrhiza*	594.75 Mb	2.7 Mb	32 483	S	2020	Phenolic acids and tanshinones	[54]
*Salvia splendens*	809.16 Mb	3.77 Mb	88 489	C	2021	Anticoagulant	[101]
*Scutellaria baicalensis*	377.0 Mb	2.10 Mb	33 414	C	2020	Baicalein, scutellarein, norwogonin, wogonin	[102]
*Scutellaria barbata*	353.0 Mb	2.50 Mb	41 697	C	2020	Baicalein, scutellarein, norwogonin, wogonin	[102]
*Forsythia suspensa*	737.47 Mb	7.33 Mb	33 062	S	2020	Pinoresinol and phillyrin	[103]
*Antirrhinum majus*	520 Mb	730 kb	37 714	C	2019	Antirrhinoside and linolenic acid	[104]
*Chimonanthus praecox*	695.36 Mb	2.19 Mb	23 591	C	2020	Essential oil	[105]
*Cinnamomum kanehirae*	730.42 Mb	498.9 kb	27 899	C	2019	Camphor	[106]
*Paris polyphylla*	70.18 Gb	1.81 kb	34 257	S	2020	Polyphyllins	[107]
*Ricinus communis*	325.5 Mb	21.1 kb	31 237	S	2010	Ricin	[108]
*Hypericum perforatum*	373.65 Mb	1.41 Mb	29 150	S	2021	Hypericin, hyperforin, and melatonin	[109]
*Linum usitatissimum*	318.25 Mb	20.1 kb	43.484	S	2012	Unsaturated fatty acids and secoisolariciresinol diglucoside	[110]
*Passiflora edulis*	1.34 Gb	6.40 Mb	23 171	C	2021	C-glycosyl flavonoids	[111]
*Aquilaria sinensis*	783.8 Mb	60.21 Kb	35 965	C	2020	Sesquiterpenes and flindersiachromone	[112]
*Punica granatum*	328.38 Mb	66.97 kb	29 229	C	2017	Punicalagin	[113]
*Euryale ferox*	725.2 Mb	4.75 Mb	40 297	C	2020	Sterols	[77]
*Nymphaea colorata*	409 Mb	2.1 Mb	31 580	C	2020	Alkaloids	[114]
*Aristolochia contorta*	210.53 Mb	2.63 Mb	18 311	C	2022	Benzylisoquinoline alkaloids and aristolochic acids	[115]
*Piper nigrum*	761.22 Mb	NA	63 466	C	2019	Piperine	[116]
*Coix lacryma-jobi*	1.73 Gb	3.19 Mb	44 485	C	2020	Coixol	[117]
*Nelumbo nucifera*	821.29 Mb	484.3 kb	32 124	C	2020	Neferine, liensinine, isoliensinine, and nuciferine	[118]
*Macleaya cordata*	378 Mb	25 kb	22 328	S	2017	Sanguinarine and chelerythrine	[119]
*Papaver somniferum*	2.72 Gb	1.77 Mb	51 213	C	2018	Morphine and codeine	[49]
*Coptis chinensis*	936.60 Mb	806.6 kb	41 004	C	2021	Protoberberine-type alkaloids	[120]
*Cannabis sativa*	876.15 Mb	1.96 Mb	33 674	C	2018	Delta-9-tetrahydrocannabinolic acid and cannabidiolic acid	[121]
*Broussonetia papyrifera*	386.83 Mb	171.2 kb	30 512	C	2019	Saponin, vitamin B and oil	[122]
*Morus notabilis*	335.39 Mb	5.72 Mb	26 010	C	2020	Phenolic acids, flavonoids and alkaloids	[123]
*Ziziphus jujuba*	437.65 Mb	34.0 kb	32 808	C	2014	Ziziphus saponin	[124]
*Eriobotrya japonica*	760.10 Mb	5.02 Mb	45 743	C	2020	Volatile oil, amygdalin and ursolic acid	[125]
*Rosa chinensis*	513.85 Mb	22.2 Mb	36 377	C	2018	Gallic acid	[126]
*Boehmeria nivea*	344.62 Mb	24.5 kb	30 237	S	2018	Ramie acid	[127]
*Pistacia vera*	671.28 Mb	714.2 kb	31 784	S	2019	Phenolic compounds and vitamins	[128]
*Acer truncatum*	633.28 Mb	773.17 kb	28 438	S	2020	Unsaturated fatty acids (Nervonic acid)	[129]
*Xanthoceras sorbifolium*	439.97 Mb	645.45 kb	21 059	C	2019	Unsaturated fatty acids (Nervonic acid)	[130]
*Rhodiola crenulata*	344.5 Mb	25.4 kb	31 517	S	2017	Salidroside and tyrosol	[131]
*Ipomoea nil*	734.8 Mb	1.87 Mb	42 783	C	2016	Diterpenoids	[132]
*Datura stramonium*	1.29 Gb	58.2 kb	30 934	S	2021	Scopolamine and atropine	[133]
*Wurfbainia villosa*	2.80 Gb	9.13 Mb	42 588	C	2022	Bornyl acetate, borneol, and camphor	[134]
*Zingiber officinale*	1.53 Gb	4.68 Mb	39 217	C, H	2021	Gingerols, gingerdiols, zingerone, paradols, and shogaol	[135]
*Selaginella tamariscina*	300.73 Mb	2.14 kb	27 761	S	2018	Selaginellins and amentoflavone	[136]
*Ginkgo biloba*	9.87 Gb	1.58 Mb	27 832	C	2021	Ginkgolide and bilobalide	[137]
*Gnetum montanum*	4.07 Gb	475.2 kb	27 491	S	2018	Stilbenoids	[138]
*Taxus chinensis*	10.23 Gb	2.44 Mb	42 746	C	2021	Paclitaxel	[139]
*Taxus wallichiana*	10.9 Gb	8.6 Mb	44 008	C	2021	Paclitaxel	[55]

a Genome assembly level.

C, chromosome level; S, scaffold level; H, haplotype-resolved genome assembly; NA: not reported

The chromosome-level genome assemblies are instrumental to highly robust downstream genomic analyses such as comparative genome analysis, chromosome evolution analysis, and gene cluster characterization etc. For example, chromosome-level assemblies of *P. somniferum* has allowed Guo *et al.* to discover a gene cluster (BIA gene cluster) that encodes biosynthetic pathways for two morphinans: morphine and noscapine [49]. Two additional assemblies of *Papaver* species produced by Yang *et al.* have enabled them to reconstruct the evolutionary history of *Papaver* karyotypes, showing morphinan biosynthetic pathways underwent punctuated evolution pattern [57]. Tu *et al.* [53] assembled a high quality chromosomal-scale *Tripterygium wilfordii* genome and found the recent duplication of triptolide biosynthetic pathway genes. Then multiple omics methods were integrated to construct gene-to-metabolite network, and finally a CYP728B70 that participated in triptolide biosynthesis was identified. The genome assemblies for *C. acuminata* [50] and *Catharanthus roseus* [33] also provide comprehensive genomic resources for the analysis of camptothecin and vinblastine biosynthesis pathway, which share common upstream to produce loganic acid, and then flux into two independent branches, respectively. The *C. roseus* genome, coupled with chemical investigations, enabled the discovery of the last two enzymes of precondylocarpine acetate synthase and dihydroprecondylocarpine synthase responsible for vinblastine biosynthesis [33, 58], resolving a long-standing question of how vinblastine/vincristine is synthesized, making their heterologous production possible. The *C. acuminata* genome found two secologanic acid synthases that converted the loganic acid flux into camptothecin production, and the downstream candidate genes set the foundation to fully discover camptothecin biosynthetic mechanism [50]. Furthermore, high quality genomes are also essential in genome-wide association studies (GWAS) to identify quantitative trait loci (QTLs) associated with PNP content. For example, Fan *et al.* assembled a chromosome-level genome of medicinal plant *Panax notoginseng* and performed GWAS on 240 cultivars to identify genes linked to dry root weight and stem thickness [59]. The availability of high-quality reference genomes provides critical resources to the elucidation of biosynthetic pathways of high-value PNPs in medicinal plants.

## Discovery of metabolic gene clusters through genomic mining

Microbial genes controlling secondary metabolite biosynthesis are typically clustered in certain genomic regions, known as metabolic gene clusters (MGCs). Unlike microbes, plant biosynthesis genes are usually dispersed throughout the genome and MGCs have long been considered rare in plants. However, MGCs have recently been identified in several plants including Arabidopsis [140, 141], rice [142], maize [143], and several medicinal plants such as opium poppy [49] and *Taxus* [55, 139]. The MGCs contain at least three non-homologous genes, ranging from tens to several hundred kilobases in total length. To date, over 30 MGCs in plants have been reported to encode PNP biosynthetic pathways based on experimental evidence [143], encoding terpenoides [140–142], alkaloids [49], steroidal glycoalkaloids [144], and fatty acids [145]. Some of these PNPs encoded by gene clusters have medicinal values such as morphine and noscapine, while many have known roles in antimicrobial, allelopathic activity, and plant defense against herbivores and pathogens. Many of these functionally characterized MGCs have been discovered even before a reference genome is available. In the post-genomic era, the availability of reference genomes expedited identification of MGCs in plants using genomic mining. Recently, computer algorithms such as Plantismash [146], Phytoclust [147] and PlantClusterFinder [148] have been developed to predict MGCs encoding potential biosynthetic pathways of secondary metabolites. A large number of potential MGCs have been found in plants encoding unknown biosynthetic pathways, highlighting the limitation of our knowledge of what and how plants can produce chemically. For example, genome mining of *P. somniferum* genome has revealed 84 MGCs, among which one cluster encoding the pathway for morphinan and noscapine has been functionally validated [49, 149]. Tomato (*Solanum lycopersicum*) has 47 predicted MGCs, four of which have been associated with alpha-tomatine [144], lycosantalonol [150], fatty acids [145], and hydroxycinnamic acid amide [151] biosynthesis. A six-gene cluster for taxadiene biosynthesis was recently identified in the *Taxus* genome, involving in the first two biosynthetic steps, which helps to decode the complete taxol biosynthesis in the future [139]. Wheat genome mining combined with transcriptomic analysis has recently identified six pathogen-induced biosynthetic pathways encoded by MGCs, producing flavonoids and terpenes that could potentially be used as phytoalexins in disease control [152]. Combining transcriptomic and metabolic profiling data would be useful to link MGCs with particular metabolites, such as the targeting of a four-gene cluster with the falcarindiol biosynthesis in tomato [145], although such strategy faces the challenge of lacking co-expression in many MGCs.

Despite the genomic discovery of hundreds of plant MGCs, questions remain to be answered regarding this specialized genetic architecture. First, how did plants gain gene clusters during evolution? MGCs in bacterial and fungi are commonly formed through gene duplication, translocation, and horizontal gene transfer (HGT). Despite a few exceptions [153, 154], it is uncommon for plants to undergo HGT and there must be special mechanisms for MGC formation in plants. Recent genomic analyses [57, 155] have shown that structural variation events such as whole genome duplications, chromosome fission and fusion, gene duplication, translocation and loss have been implicated in the birth and evolution of biosynthesis gene clusters, supporting the theory of punctuated evolution in forming plant specialized metabolites. Overall, investigation of plant MGC evolution remains at the infant stage, requiring comparative analysis of a large number of plant genomes. It will help us understand the major driving forces of PNP evolution and its ecological impact in nature.

Second, how do many of the MGCs actually contribute to PNP biosynthesis? As more candidate MGCs continue to be identified from plant genomes via an *in silico* approach, it is critical to functionally characterize predicted MGCs of unknown function and associate them with potential natural products. Expressing the candidate MGCs in a heterologous host such as yeast or *E. coli* will be informative to determine the synthesized product, as shown by several recent examples [151, 156]. For instance, Kong *et al.* used yeast as a chassis to investigate the function of a tomato gene cluster, and discovered a novel naringenin chalcone synthase responsible for the production of dihydro-coumaroyl anthranilate amide [151]. The caveat of this approach is that most predicted plant MGCs are quite large (up to hundreds of kb), presenting a huge challenge to cloning them into bacterial or yeast expression vectors. The plant MGCs successfully cloned and expressed in microbial hosts are mostly mini gene clusters of several kb containing only a few open reading frames. In addition, expressing plant proteins in yeast or *E. coli* cells does not always work due to different codon usage and post-translational modifications in eukaryotic versus prokaryotic cells. Alternatively, validation using plant host such as *Nicotiana benthemiana* enables expression of candidate MGCs via *Agrobacterium* infiltration of plant leaves or cells, followed by metabolomic detection, although it faces the same problem of delivering and expressing long MGC fragment into tobacco cells. Recently, a platform for high-throughput secondary metabolite discovery has been developed for filamentous fungi by cloning and expressing fragmented genomic DNAs containing MGCs into fungal artificial chromosomes followed by metabolomic profiling [157]. Application of this or a similar approach has not been reported in plants considering the low genomic fraction of plant MGCs and lack of a proper artificial chromosome cloning system. A high-throughput MGC validation platform will expedite the identification and utilization of novel PNPs.

## Bioengineering of PNP through plant biotechnology

In nature, PNPs are accumulated at low abundance and only in specific tissues and developmental stages of medicinal plants for two major reasons. Firstly, biosynthesis of these compounds consumes energy and competes with normal plant vegetative growth and reproduction. Secondly, most PNPs have cell toxicity from which plants have to protect themselves by detoxification, storing them in compartments, or only producing the toxic chemicals when and where needed. Evolutionarily, PNPs have probablyundergone natural as well as human selection. For example, *P. somniferum* accumulates high levels of painkilling morphines in capsules instead of other tissues, and the amount of morphine produced differed among cultivars [158]. By contrast, its close relative *Papaver rhoeas* only produces a trace amount of morphine [57]. This suggests that the ability to produce morphine has been under natural selection in poppy plants, and likely selected by domestication and breeding process.

The naturally low content of PNPs in medicinal herbs renders a major bottleneck in drug developments and clinical therapeutics. Structures of PNPs are often too complex for a profitable production by total chemical synthesis. Therefore, plant extraction remains the primary commercial source of most PNP for pharmaceuticals, causing over-exploitation of natural resources and instability to the Earth’s ecosystem. For instance, taxol, a well-known anticancer drug ingredient derived from the bark of yew tree once put the yew on the verge of extinction due to exhaustive exploitation. The demand of medical PNPs thus stimulates the breeding of superior germplasm resources for sustainable use of medicinal herbs. There are many challengesin breeding medicinal plants for high PNP yield, including limited understanding of how PNPs are exactly made and regulated by plants, lack of high-quality genome sequence, annotation and molecular markers, long breeding cycles as well as the difficulty of genetic transformation. Herb genomic research has accelerated the identification of functional genes and genome-wide molecular markers, linked molecular markers with desired characters, and improved breeding medicinal herbs. Many efforts have been made to increase PNP yield through plant breeding and biotechnological improvement, including artemisinin in *A. annua* [159, 160], morphine in *P. somniferum* [161], THC (tetrahydrocannabinol) in *Cannabis sativa* [162], etc. *A. annua* has been a primary source of the anti-malaria drug artemisinin. *A. annua* transcriptome sequencing enabled construction of genetic linkage map and identification of quantitative trait loci (QTL) that control artemisinin yield [160], providing genetic resources for molecular breeding. Phenotype selection coupled with molecular breeding in the past decadehas led to the production of Artemisia F1 Seed (https://www.artemisiaf1seed.org), increasing the artemisinin yield from 5 kg per hectare to 55 kg per hectare with a 1.44% of dry weight [159]. To date, *A. annua* remains the sole source of artemisinin globally, although artemisinin metabolic engineering has been reported [163].

The rise of modern biotechnology provides a novel strategy for precise and expedited medicinal plant breeding. A key breakthrough is the revolutionary genome editing technology, most notably the CRISPR-cas9 system, that allows precise genome bases to obtain traits of interest at an unprecedented pace [164]. This biotechnology has powered the next generation ofplant breeding to improve crop yield and quality such as PNP content. Unlike model and crop plants, the genome editing of medical herbs is still at the infant stage, hindered by the lack of genomic information and a reliable genetic transformation system. Nevertheless, CRISPR-Cas9 based genome editing has been reported in several medicinal plants towards optimizing production of pharmacological components in *P. somniferum* [165], *S. miltiorrhiza* [166], *Dendrobium officinale* [167] and *Camelina sativa* [168]. Notably, genome editing successfully targeted three *FAD2* (fatty acid desaturase 2) genes in allohexaploid *Camelina sativa* and enhanced seed fatty acid levels [168]. CRISPR-Cas9 mediated gene deletion significantly decreased the benzylisoquinoline alkaloid flux in transgenic opium poppies [165]. Plant genetic engineering offers clear advantages in improving the yield of PNPs [169] as plant chassis naturally carries many fundamental plant biosynthetic gene circuits, making them natural cell factories to produce PNPs of interest. A paradigmatic case is the Golden Rice [170], where the whole β-carotene biosynthetic pathway was introduced into rice endosperm using *Agrobacterium*-mediated co-transformation to generate rice plants with carotenoid content up to 1.6 mg/g in the endosperm. Moreover, a high-efficiency vector system was developed for transgene stacking to engineer anthocyanin biosynthesis in rice endosperm [171]. In addition, Zhu *et al.* [172] developed a novel method called combinatorial nuclear transformation to generate multiplex-transgenic plants allowing five carotenogenic genes to be simultaneously transferred into a white maize through biolistic transformation, resulting in transgenic plants with elevated levels of β-carotene.

Besides modifying existing metabolic pathways, genetically engineered plant chassis offers a cheap and sustainable source to produce high-value PNPs. The *de novo* production of PNPs in model organisms such as *Arabidopsis*, tobacco, tomato, and moss has progressed rapidly recently. For example, Fuentes *et al.* [173] transferred the entire artemisinic acid metabolic pathway from *A. annua* to tobacco chloroplast genome using combinatorial supertransformation of transplastomic recipient lines (COSTREL). Plants with high artemisinic acid levels were then isolated through screening large populations of transplastomic lines. In addition, strategies to increase terpenoids including overexpression of rate-limiting enzymes, chloroplast-compartmentalized engineering, and integration of transcription factors have been applied to the production of momilactone [174] and taxadiene [175] in tobacco. Momilactones are a group of diterpenes predominantly found in rice with an allelopathic activity. Through changing the subcellular localization of prenyltransferase and diterpene synthases, the diterpene biosynthesis was rerouted from chloroplast to cytosolic MEP pathway, significantly promoting the production of momilactone in *N. benthamiana* [175]. Noteworthy, this strategy also enabled the discovery of missing steps in momilactone B pathway, providing insights into pathway reconstitution and elucidation for desired products.

Metabolic engineering of an *in vitro* plant tissue culture, such as suspension cell culture and hairy root culture, is another efficient approach to yield valuable phytochemicals. Hairy roots are induced by *Agrobacterium rhizogenes* mediated transformation, which can be applied as high-capacity bioreactor to produce PNPs without the need of light and hormones. Hairy root cultures were successfully induced to overproduce cannabinoids in *C. sativa* [176], phytosterols and ginsenosides in *Panax ginseng* [177], and curcumin in *Atropa belladonna* [178]. Suspension cell culture has also made great advances to produce valuable PNPs with high yields. Plant Cell Fermentation (PCF®) Technology (https://phytonbiotech.com/) could produce natural taxol directly from plant cells of *Taxus chinensis* v. *marei*, while the metabolic engineered grapevine cells were able to produce resveratrol derivatives when elicited with MeJA and methylated cyclodextrins [179]. In the suspension cell culture system, the addition of heterologous elicitors induces the biosynthetic gene expression and increases the production of PNPs. Glandular trichomes are hairy structures differentiated from epidermal cells, featured by their enormous capacity to synthesize, store and secret large quantities of metabolites with distinct types, making them an excellent platform for decoding the biosynthesis pathway of PNPs, and efficient phytochemical factories to produce PNPs [180]. Kortbeek *et al.* [181] engineered tomato glandular trichomes where a farnesyl diphosphate synthase was overexpressed, resulting in a decline of monoterpenoid production in the trichomes.

## Synthetic biology: a green revolution for PNP bioengineering and industrialization

Although plant breeding can produce cultivars that accumulate higher level of metabolites than others do, it is still inefficient and environmentally unsustainable for massive production for commercial uses in most cases. Alternatively, synthetic biology where microbial (e.g. yeast and bacteria) chassis are used to massively produce PNPs offers a more effective and environment-friendly alternative. Synthetic biology aims to design microbial cell factories carrying genetic circuits made of biosynthetic genes for heterologous production of PNPs. It has several advantages over plant-based extraction, such as circumventing the requirement of growing plants, rapid production and little interference from natural environment [182]. With the development of synthetic biology tools and knowledge of biosynthetic pathways, PNPs such as artemisinic acid [183], amorphadiene [163], taxadiene [36], cannabinoids [184], morphine [38], and noscapine [185] has been successfully produced using engineered microbial cells. A prominent example is the semi-synthesis of artemisinin [183], where an engineered amorphadiene-producing yeast [38] produces artemisinic acid with the titer of 25 g/L, later converted to artemisinin by chemical synthesis. This opens a route to the industrial production of artemisinin against the urgent demand of anti-malarial drugs. Parallelly, Luo *et al.* [184] partitioned the cannabinoid metabolic pathway into three modules: an engineered *S. cerevisiae* MEP pathway to make more flux into geranyl pyrophosphate, a hexanoyl-CoA biosynthetic pathway and several *Cannabis* genes to accumulate more olivetolic acid, as well as a heterologous downstream pathway to form the corresponding cannabinoids. The engineered yeast strains yielded 1.6 mg/L of cannabinoid from the simple sugar galactose, laying a foundation for the large-scale production of cannabinoids.

**Figure 3:**
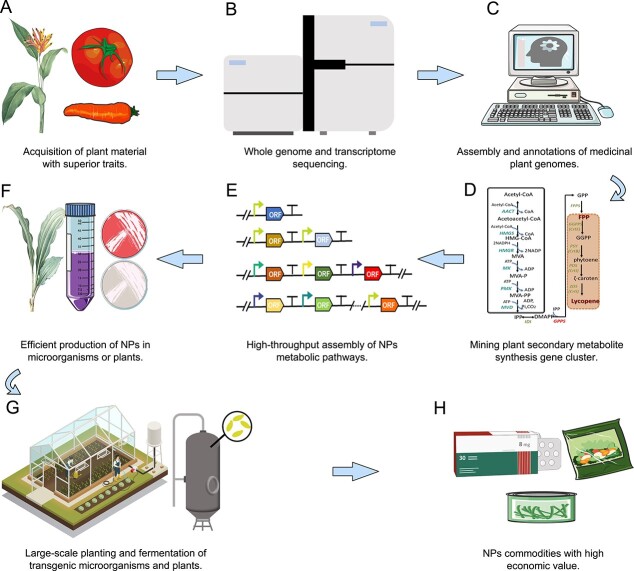
A roadmap for green revolution of herb medicine: a typical workflow for plant natural product (PNP) production via synthetic biology. **A** Selecting plants with superior PNP traits using traditional and transgenic breeding methods. **B** Whole genome and transcriptome sequencing of plant samples. **C** Assembly and annotations of medicinal plant genomes. **D** Revealing plant secondary metabolite synthesis gene cluster by omics data mining. **E** High-throughput assembly of PNP metabolic pathways. **F** Efficient production of PNPs in microorganisms or plants. **G** Industrial-scale planting and fermentation of transgenic microorganisms and plants. **H** Making PNP commodities with high commercial value.

Despite progress, it remains challenging to use microbe as a chassis to synthesize high-value PNPs [182]. First, decoding biosynthetic pathways is the prerequisite for successful heterologous production but remains a challenge for vast majority of PNPs. For instance, previous attempts to reconstruct taxol pathways in microbe without knowing the steps converting downstream taxadiene to final taxol ended up with a production of taxadiene, the first committed intermediate [185]. The combination of bioinformatics and downstream functional validations in heterologous hosts to resolve the biosynthetic pathways of PNPs from sequencing data plays a key role in synthetic biology today (Fig. 3). For example, the *Taxus* genome analysis identified a functional grouping of CYP725As and a taxadiene gene cluster, which will facilitate the future elucidation of taxol biosynthesis [55, 146].

Second, choosing and optimizing a microbial host is essential to maximize yield of PNPs. *E. coli* and *S. cerevisiae* are two of the most widely used microorganisms to engineer biosynthetic pathways, given their fast growth, well-known genetic background and well-established genetic manipulation methods [36, 38, 163, 183–185]. However, it is challenging to express functional plant-derived cytochrome P450 genes in prokaryotic *E. coli* which lacks an intracellular organelle system, post-translational modification and electron transfer machinery, thus limiting its application in heterologous production of many PNPs [36]. For example, *E. coli* was engineered to produce artemisinic acid with high titer of 20 g/L amorphadiene, achieving only 1 g/L artemisinic acid [186], much lower than the aforementioned 25 g/L artemisinic acid in *S. cerevisiae*. Apart from the commonly used *E. coli* and *S. cerevisiae*, nonconventional chassis cells are also used such as the industrial production of 4-hydroxybenzoic acid by *C. glutamicum* [187] and (+)-nootkatone by *Pichia pastoris* [39].

Third, reconstituting an efficient module is key to heterogeneous production of PNPs. To construct heterogeneous expression cassettes, codon-optimization of plant genes is often required for microbial expression [36, 38]. Partition of complex biosynthetic pathways into several modules [36, 184] and the strains are optimized by synthetic biology tools.

Finally, the balance between microbial growth and PNP yield should be considered as many PNP and their intermediates, such as alkaloids and phenols, are toxic to the microorganisms. Taken together, with the rapid development of technologies including genome sequencing, multi-omic data mining, structure biology, directed evolution of proteins, and design of novel proteins, it is expected that the efficiency in synthesizing PNPs in microorganisms will be greatly improved in the near future.

**Table 2:** A summary of transcription factors (TFs) regulating biosynthesis of plant natural products (PNP).

PNP types	Species	TFs	Regulated genes	PNPs
Flavonoids	*Citrus reticulata* cv. *suavissima*	CitERF32 [188], CitERF33 [188]	*CitCHIL1*	Naringenin chalcone, (2S)-Naringenin
	*Antirrhinum majus*	Delila [189], Perilla [189]	*CHS*	Red anthocyanin
	*Gentiana triflora*	GtbHLH1 [190]	*GtF3’5’H*, *Gt5AT*	Gentiodelphin
	*Petunia hybrida*	AN1 [191]	*dfrA*, *Pmyb27*	Anthocyanin
	*Myrica rubra*	MrbHLH1 [192]	*MrCHI*, *MrF3’H*, *MrDFR1*, *MrANS*, *MrUFGT*	Anthocyanin
	*Epimedium sagittatum*	EsMYBF1 [193]	*EsCHS, EsCHI, EsF3’H, EsF3H*, *EsFLS*	Anthocyanin, Kaempferol, Andquercetin
	*C. reticulata*	CsMYBF1 [194]	*Cs4CL, CsCHS*, *CsFLS*	Hydroxycinnamic acid
	*Ginkgo biloba*	GbMYBF2 [195]	*GbCHS, GbF3H GbPAL*, *GbFLS*, *GbANS*, *GbCHI*	Quercetin, Kaempferol, Anthocyanin
	*P. hybrida*	AN2 [191], AN4 [191]	*dfrA*, *Pmyb27*	Anthocyanin
	*M. rubra*	MrMYB1 [192]	*MrCHI*, *MrF3’H*, *MrDFR1*, *MrANS*, *MrUFGT*	Anthocyanin
	*A. majus*	AmMYB305 [196], AmMYB340 [197], Rosea1 [198], Rosea2 [198], Venosa [198]	*PAL*, *CHI*, *F3H F3′H*, *FLS*, *DFR*, *UFGT*, *ANS*	Magenta anthocyanin
	*Gentiana triflora*	GtMYB3 [197]	*GtF3’5’H*, *Gt5AT*	Gentiodelphin
	*Ipomoea nil*	InMYB1 [199]	*CHS-D*, *CHI*, *F3H*	Anthocyanin
	*Medicago truncatula*	MTWD40–1 [200]	*DFR*, *ANS*, *LAR*, *ANR*, *UGT72L1*, *CHS*	Anthocyanin, Proanthocyanidin, Benzoic acids, Flavonols, Flavan-3-ols
	*I. nil*	InWDR1 [199]	*CHS-D*, *CHI*, *F3H*	Anthocyanin
Terpenoids	*Artemisia annua*	AaERF1 [201], AaERF2 [201], AaORA [202], TAR1 [203]	*ADS*, *CYP71AV1*, *DBR2*, *AaERF1*	Artemisinin, Artemisinic acid, Dihydroartemisinic acid
	*Panax ginseng*	ERFs [204]	*DDS*, *CYP716A47*, *CYP716A53v2*	Ginsenoside Rb1, Ginsenoside Rg1, Ginsenoside Re
	*A. annua*	AabZIP1 [205]	*ADS*, *CYP71AV1*	Artemisinin
	*A. annua*	AaWRKY1 [206,207]	*ADS*, *CYP*	Artemisinin
	*A. annua*	AaNAC1 [208]	*ADS*, *DBR2*, *ALDH1*	Artemisinin, Dihydroartemisinic acid
	*A. annua*	AabHLH1 [209]	*HMGR*, *ADS*, *CYP71AV1*	Artemisinin
	*P. ginseng*	PGbHLHs [210]	*DXS*, *DXR*, *IspF*, *IspG*, *IspH*, *ACTT*, *HMGS*, *HMGR*, *MVK*, *PMK*, *FPS*, *SS*, *SE*, CAS, OAS, *PPDS*, *PPTS*, *DDS*, *β-AS*	Ginsenosides
	*Glycyrrhiza uralensis*	GubHLH3 [211]	*β-AS*, *CYP93E3*, *CYP72A566*	Soyasapogenol B, Sophoradiol
	*Betula platyphylla*	BpbHLH9 [212]	*HMGR*, *FPS*, *SS*, *SE*, *BPY*, *BPW*	Betulinic acid, Oleanolic acid, Betulin
	*M. truncatula*	TSAR1 [213], TSAR2 [213]	*HMGR1*, *MAKIBISHI1*	Hemolytic saponin, Nonhemolytic soyasaponi
	*A. annua*	AaEIN3 [214]	*ADS*, *DBR2*, *CYP71AV1*, *AaORA*	Artemisinin
	*A. annua*	AaMYB1 [215]	*FDS*, *ADS*, *CYP71AV1*	Artemisinin, Dihydroartemisinic acid
	*P. ginseng*	PgMYB2 [216]	*PgDDS*	Ginsenoside
	*Mentha spicata*	MsMYB [217]	*MsGPPS.LSU*	Terpene
	*Salvia miltiorrhiza*	SmMYB36 [218], SmMYB9b [219]	*DXS1, DXS2, DXR, MCT, MDS, HDS, CMK, HDR1, GGPPS1, CPS1, KSL1, CYP76AHI, SmDXS2, SmDXR, SmGGPPS, SmKSL1*	Phenolic acids, Tanshinones
	*Glycine max*	GmMYBZ2 [220]	*ZCT1*, *ASα*, *STR*, *ORCA3*	Catharanthine
	*Pogostemon cablin*	PatSWC4 [221]	*PatPTS*	Patchoulol
	*P. cablin*	PatTHs [222], PatGT-1 [222]	*PatHMGR*	Patchoulol
	*Salvia miltiorrhiza*	SMJAZ1/2/3/9 [223, 224]	interaction with MYC2	Tanshinones, Lithospermic acid B
	*M. spicata*	MsYABBY5 [225]	*MsWRKY75*	Monoterpene (limonene and carvone)
Alkaloids	*Eschscholzia californica*	20 EcAP2/ERF TFs [226]	*Ec6OMT*, *EcCYP719A5*	Benzyl isoquinoline alkaloids
	*Catharanthus roseus*	ORCA1/2/3 [227–229], CrERF5 [230], CrCR1 [231]	*STR*, *ASα*, *TDC*, *CPR*, *DXS*, *D4H*, *SLS*, *GES*, *SLS1*, *SGD*, *Redox1*, *SAT*, *PRX1*, *HL1*, *G10H*, *DAT*	Vindoline, Catharanthine, Vinblastine, Vincristine, Secologanin, Ajmalicine, Anhydrovinblastine, Serpentine
	*Nelumbo nucifera*	NnbHLH1 [232]	*NnNCS1*	Benzyl isoquinoline alkaloid
	*Coptis japonica*	CjbHLH1 [233]	*TYDC, NCS, 6OMT, CNMT, CYP80B2, 40OMT, BBE, SMT, CYP719A1*	Isoquinoline alkaloid
	*C. roseus*	CrMYC1, CrMYC2, CrBIS1, CrBIS2, CrBIS3 [234–236]	*TDC*, *STR*	Terpenoid indole alkaloid
	*C. roseus*	ZCT1 [237], ZCT2 [237], ZCT3 [237]	*SSR2*, *C5-SD*, *HMGR, CAS*	Steroidal glycoalkaloids
	*Coptis japonica*	CjWRKY1 [238]	*TYDC*, *NCS*, *6OMT*, *CNMT*, *CYP80B2*, *40OMT*, *BBE*, *CYP719A1*, *SMT*	Berberine
	*C. roseus*	CrWRKY1 [239]	*TDC*, *AS*, *DXS*, *SLS*, *SGD*	Terpenoid indole alkaloid
	*C. roseus*	CrGBF1 [240], CrGBF2 [240]	*STR*	Terpenoid indole alkaloid
	*C. roseus*	CrGATA1 [241]	*T16H2*, *D4H*, *DAT*, *T3O*, *T3R*	Vindoline, Tabersonine
	*C. roseus*	CrBPF1 [242]	*D4H*, *DAT*	Terpenoid indole alkaloid
	*N. nucifera*	NnMYB6 [232], NnMYB12 [232], NnMYB113 [232]	*NnTYDC1*, *NnCYP80G*, *Nn7OMT2*	Benzyl isoquinoline alkaloid
	*N. nucifera*	NnRAV1 [232]	*NnTYDC1*, *NnNCS1*	Benzyl isoquinoline alkaloid

## Opportunities and challenges for future PNP research

It is an exciting time to study PNP in this golden era of chemical biology. New technologies in both experimental and computational sciences are emerging at an unprecedented pace. These technologies have enabled the untangling the complex biosynthetic pathways and networks using systems biology approach, providing insight into the mechanistic and evolutionary mechanisms behind the chemical and structural diversity of natural products. Accurate and complete assembly of medicinal plant genomes is the key to understanding genome function and evolutionary patterns and improving plant traits through breeding and synthetic biology. Horticultural plant genomes are notoriously difficult to assemble, due to their wide range of genome sizes (up to hundreds of Gb), various ploidy levels, high repeat content and heterozygosity. Yet with genome technologies advancing quickly in recent years, chromosome-scale assembly, once a rarity, is now a realistic target for most genome projects. A typical genome project now adopts a combination of different technologies including long-read and short-read sequencing data, 10× Genomics data, Hi-C sequencing data or Bionano optical genome maps. Bioinformatic algorithms are continuing to be developed to solve complex problems of genome assembly and annotation leveraging these different technologies [243]. As a result, a growing number of medicinal plants now have chromosome-level genome assembly and some offer obvious improvement over previous versions of assembly and annotation [244, 73].

Despite the progress, a long journey is ahead to resolve the complete genome sequence of medicinal plants. Twenty years after the first plant reference genome was produced, the majority of plant reference genomes initially assembled using short reads remain unimproved. Recently, a telomere to telomere (T2T) genome assembly has been produced for a human cell line CHM13, resolving the nearly complete haploid genome sequence of human being [245]. The achievement is largely reliant on the use of high fidelity PacBio long-read sequencing data (HiFi reads), in addition to the ultra-long ONT reads and Hi-C reads. This is not just a milestone of human genomics, but also has a profound impact on animal and plant genomic research by kicking off an ambitious journey to resolve the complete genome sequence of all known living organisms on this planet. In fact, shortly after the publication of the human T2T genome assembly, the nearly complete genome sequence of *A. thaliana* [246, 247], and the gap-free reference genomes of rice [248] and watermelon [249] were reported. The nearly complete genome sequences of plants reveal tandem repeats of satellite regions located in centromeres, and find new genes that weren’t accessible in previous versions of assemblies. T2T plant genome sequences will make huge impact on plant biology allowing researchers to grasp a full complement of genetic elements associated with various traits including growth, development, and physiology. Another technical challenge for plant genome assembly is, instead of generating a collapsed diploid assembly, the ability to produce a haploid-resolved assembly that separates the parental and maternal haplotypes, also known as genome phasing. The phased assembly allows understanding of mechanisms behind heterosis and allele-specific gene regulation that contributes to many plant biological processes. To date there are only a handful of plant genomes that have been assembled and phased, including tea [250], lychee [251], and pear [252]. Commonly, genome phasing is conducted through trio-binning sequencing where genomes of parents are used to untangle the two haploid genomes of a child, although the information of parents is often unavailable for medicinal plants. In such cases, recently genome assembly methods such as hifiasm [253] allow for the resolution of haploids by using HiFi reads and Hi-C data without relying on sequencing data of family trios.

Unraveling the regulatory mechanisms underlying PNP biosynthesis is beneficial to improving the quality of traditional Chinese medicine through genetic breeding and metabolic engineering. Genomics and metabolomics of plant tissues have shown that the genes of biosynthetic pathways for PNPs are often co-expressed in specific tissues [49, 139]. So far it remains elusive how PNP biosynthetic enzymes and pathways are regulated in such a spatial and temporal fine-tuned manner. Studies regarding the molecular mechanisms of transcriptional regulation related to biosynthetic pathways of PNPs are still limited, most of which being focused on the specialized medicinal plants with well-known PNPs, high-quality genomic data, and an established transgenic system. The expression of PNP biosynthetic pathways is controlled by epigenetic mechanisms such as chromatin topology dynamics as shown by Nützmann *et al.* in model plant Arabidopsis [254]. Thus, it will be exciting to reveal what roles 3D genomic architecture and organization play in regulating biosynthesis of flavonoids, terpenoids, and alkaloids in medicinal plants. In addition, several transcription factors have been identified to regulate the process over the years (Table 2), offering targets to enhance PNP accumulation potentially via overexpression (activator) or silencing (repressor). However, it remains a challenge to stably transform most medicinal plants and obtaining transgenic plants often takes years even if it does show promise of significantly improving the PNP level. Another caveat is that expressing an excess amount of any epigenetic or transcriptional regulators, usually entangled in complex regulatory networks, can potentially lead to undesired traits. Therefore, a systems biology approach is essential to tease apart the PNP-specific gene circuits for precision modulation of target traits. Moreover, the picture is still a blur in cell heterogeneity that accounts for the cell-type specific expression of biosynthetic enzymes, transporters, and gene regulators. Recently, cutting-edge technologies such as single-cell transcriptomics (scRNA-seq) and spatial-transcriptomics have been widely applied to mammalian and plant tissues to generate a cell atlas and identify cell types within these tissues. The use of such technologies in PNP research has yet to be reported as of the date of thisreview being written, although spatial metabolomics has been reported to investigate the distribution of plant metabolites [255, 256]. It will be very interesting to identify the specific cell-types expressing PNP biosynthetic genes using the scRNA-seq and spatio-transcriptomics, guiding a precise design and bioengineering of gene circuits for PNP improvement. The key challenge of the application of single-cell genomics in medicinal plants includes lack of high-quality reference genome and gene annotations, single-cell preparations, tissue and cell-type specific markers, and robust methods to integrative anlaysis of omic profiles at a single-cell level. Protocols and methods developed for human and mammalian samples are well in place but remain to be tested and optimized for medicinal plant studies.

Last but not least, plant and microbe interactions also have a big influence on the profiles and abundance of PNPs. The importance of location and environment for growing TCM to their medicinal values and pharmaceutical properties has long been recognized and documented in traditional medicine scriptures. The difference could be down to a combination of factors such as the ecological environment, weather and perhaps the microbiota inhabiting in the soils where the TCM grows. Although the exact formula of microbial communities involved in modulating the PNP, as well as the mechanisms of regulation remain elusive, the association between microbiota and medicinal properties in plants has been suggested in several recent studies. An outstanding example of such association is reported in model plant *A. thaliana* which produces specific types of triterpenoids to electively modulate root bacteria in root microbiome [257]. Interestingly, isolation and re-inoculation of these bacteria in the roots can induce an increased production of these very PNPs. It remains to be studied how different types of microbes form microbial communities and signaling networks to interact with plants, either internally (endophytes) or externally (*e.g.* surface of roots and leaves), contribute to the accumulation of specific PNPs. Equally interesting is the regulation of plant microbiota by plant metabolites during plant–microbe interactions. Compared to model and crop plants, microbiome studies of medicinal plants have been quite limited. A combination of culture-based and culture-free microbiome analysis with functional metabolomics in medicinal plant rhizosphere and endophytes will help identify the association between plant microbiota and specialized metabolites. With this knowledge, it will be possible to modulate the production of special natural products in controlled settings such as greenhouse and plant factories in the future, much more efficient and effective than that which could be harvested from traditional authentic herb medicines.

## Acknowledgements

This project is supported by the National Natural Science Foundation of China (Grant No. 31970317 and 32070368), Key Realm R&D Program of Guangdong Province, China (No. 2020B020221001), and the Natural Science Foundation of Shaanxi Province, China (No. 2020JZ-05). L.G. is also supported by a faculty startup package from Peking University Institute of Advanced Agricultural Sciences, and Yuandu Scholarship from municipal government of Weifang. The authors would like to thank anonymous reviewers for their comments and suggestions to improve the manuscript.

## Conflict of interests

The authors declare that they have no conflict of interest.
